# Influence of perceived stress on prenatal depression in Surinamese women enrolled in the CCREOH study

**DOI:** 10.1186/s12978-021-01184-x

**Published:** 2021-06-30

**Authors:** Anisma R. Gokoel, Firoz Abdoel Wahid, Wilco C. W. R. Zijlmans, Arti Shankar, Ashna D. Hindori-Mohangoo, Hannah H. Covert, Meerte-Sigrid MacDonald-Ottevanger, Maureen Y. Lichtveld, Emily W. Harville

**Affiliations:** 1 Scientific Research Center Suriname, Academic Hospital Paramaribo, Paramaribo, Suriname; 2 Department of Environmental Health Sciences, School of Public Health and Tropical Medicine, Tulane University, New Orleans, LA USA; 3 Faculty of Medical Sciences, Anton de Kom University of Suriname, Paramaribo, Suriname; 4 Foundation for Perinatal Interventions and Research in Suriname (PeriSur), Paramaribo, Suriname; 5 Department of Medical Microbiology, Academic University Medical Center, Amsterdam, The Netherlands; 6 Graduate School of Public Health, University of Pittsburgh, Pittsburgh, PA USA; 7 Department of Epidemiology, School of Public Health and Tropical Medicine, Tulane University, New Orleans, LA USA

**Keywords:** Prenatal, Probable depression, Social support, Perceived stress, Resilience, Suriname

## Abstract

**Background:**

Prenatal depression may have adverse health effects on mothers and their offspring. Perceived stress is an important risk factor for depression during pregnancy. Studies have shown that both perceived stress and depression may negatively influence birth outcomes. While 20% of pregnancies in Suriname, a middle-income Caribbean country located in northern South America, results in adverse birth outcomes, data on prenatal depression and its risk factors are lacking. This study aimed to assess the influence of perceived stress on depression during pregnancy in Surinamese women.

**Methods:**

Survey data were used from 1143 pregnant women who participated in the Caribbean Consortium for Research in Environmental and Occupational Health-MeKiTamara prospective cohort study that addresses the impact of chemical and non-chemical environmental exposures in mother/child dyads in Suriname. The Edinburgh Depression Scale and Cohen Perceived Stress Scale were used to screen for probable depression (cut-off ≥ 12) and high stress (cut-off ≥ 20), respectively. The association between perceived stress and depression was examined using bivariate and multiple logistic regression analyses, adjusted for social support (including resilience) and maternal demographics.

**Results:**

The prevalence of high perceived stress during the first two trimesters and the third trimester were 27.2% and 24.7% respectively. 22.4% of the participants had probable depression during first or second trimester and 17.6% during the third trimester. Women experiencing high stress levels during the first two trimesters had 1.92 increased odds (95% CI 1.18–3.11, *p* = 0.008) of having probable depression during the third trimester of pregnancy than those with low stress levels. Pregnant women with low individual resilience during early pregnancy (52.1%) had 1.65 (95% CI 1.03–2.63, *p* = 0.038) increased odds of having probable depression during later stages of pregnancy compared to those with high individual resilience. Low educational level (*p* = 0.004) and age of the mother (20–34 years) (*p* = 0.023) were significantly associated with probable depression during the third trimester.

**Conclusions:**

Early detection and management of stress and depression during pregnancy are important. Health education programs, targeting the reduction of stress during pregnancy, may help to reduce depression and its potential adverse health effects on the mother and child.

## Plain language summary

Depression during pregnancy may lead to adverse health effects in mothers and children. While one in five pregnancies resulted in an adverse birth outcome in Suriname, and perceived stress and depression are important risk factors for birth outcomes, data on depression and its risk factors are lacking. This study aimed to determine the association between perceived stress and prenatal depression in Surinamese pregnant women participating in the Caribbean Consortium for Research in Environmental and Occupational Health-MeKiTamara study.

A total of 1143 pregnant women were included in the study. Using questionnaires, data was collected on demographic factors, perceived stress, social support (including resilience), and probable depression.

Perceived stress was somewhat higher during the first two trimesters (27.2%), than the third trimester (24.7%). This was also the case for probable depression; higher during the first two trimesters (22.4%) than during the third trimester (17.6%). The study found a statistically significant association of high perceived stress, low perceived individual resilience, lower education and older maternal age with probable depression during pregnancy.

Early detection and effective management of perceived stress and depression during pregnancy are very important. There is a need for prenatal clinics in Suriname to routinely screen for symptoms of perceived stress and depression to minimize the potential impact on mother and child. Health education programs, targeting the reduction of stress during pregnancy, may help to reduce depression and its potential adverse health effects on the mother and child.

## Background

The prevalence of perceived stress during pregnancy ranges from 5.5 to 15% in developed countries and 33 to 52.9% in developing countries [1–3]. Possible risk factors that contribute to stress during pregnancy are: low income, lack of social support, young age (< 20 years), low socio-economic status, not married/single, parity, gravidity and low education [1, 2, 4–10]. Stress during pregnancy may induce long term adverse health effects on the mother, the unborn child and the development of the child [1, 6]. Prenatal stress is associated with lower gestational age [11], low birth weight, preterm birth [12, 13], and maternal depression [9]. Several forms of early life stress can predict elevated levels of inflammation, which in turn plays a key role in the pathogenesis of depression. Depressed adults who experienced severe forms of early life stress were more likely to have high levels of C-reactive protein (CRP) than depressed adults who did not experience these severe forms of early life stress. [14].

The prevalence of prenatal depression varies between 19 and 25% in Low- and Middle-Income Countries (LMIC) compared to 7–15% in High-Income countries [8, 15, 16]. Studies have shown the effect of intergenerational transmission of depression, where the grandmother’s depression affected the mother’s depression and her own stressful life context, and maternal and grandmother depression affected youth depression as mediated by interpersonal stress processes. In this way, depression can be passed on for generations afterwards [17, 18]. Women with prenatal depression are at higher risk for pregnancy-related complications [15] and adverse birth outcomes, e.g. low birth weight, preterm birth and intra-uterine growth restriction [8, 19]. This is also confirmed by the fetal origins hypothesis, which emphasizes the effect of environmental conditions in utero and immediately after birth on the developmental health and wellbeing of the child and the subsequent impact on adulthood e.g. inadequate nutrition in utero is associated with obesity, cardiovascular diseases and diabetes in adulthood [20]. Some risk factors for developing prenatal depression, in addition to perceived stress, are being single/unmarried [15, 21], having a low monthly income [15, 22], low education [23], race or ethnic minority (e.g. black race) [24], being a teenager, no or irregular prenatal care [15, 22], and less perceived social support [15, 22, 24]. In particular, lack of partner and family social support is strongly associated with developing depression during and after pregnancy [5, 25].

The Caribbean Consortium for Research in Environmental and Occupational Health (CCREOH)-MeKiTamara study is a prospective environmental epidemiologic cohort study which assesses the influence of non-chemical and chemical stressors on maternal and child health in Suriname. Suriname is a middle-income country situated on the northeastern coast of South America. The majority (66.3%) of Suriname’s total population resides in urban areas, while the remainder lives in rural areas (21%) and the interior rainforest (12.7%) [26]. Suriname has a multi-ethnic population consisting of Asian (41.1%; Hindustani and Javanese), African (37.4%; Tribal people and Creoles) and Other (21.0%; Mixed, Amerindians, Chinese, and Caucasians) [26]. Around 20% of all pregnancies in Suriname ends up in adverse birth outcomes [27]. The neonatal mortality rate was 12.9‰ between September 2010 and December 2012. These neonates more often had a birthweight of less than 2500 g (71.2%) and were born prematurely (67.7%) [27]. While the less favorable maternal- and child health status in Suriname may be in part attributed to prenatal depression, data on this condition and risk factors thereof are absent. The primary aim of this study was to examine the association between prenatal perceived stress on depression in pregnant Surinamese women enrolled in the MeKiTamara study.

## Methods

### Study design

The MeKiTamara study is a prospective environmental epidemiologic cohort study. MeKiTamara means “creating a mother’s and child's tomorrow” in Sranang tongo, Suriname’s lingua franca. Pregnant women were recruited during the first or second trimester of pregnancy from three regions of Suriname. The association between perceived stress and depression, adjusting for social-demographic variables, was examined using survey data.

### Study population

From December 2016 to July 2019, eligible pregnant women were recruited from (1) the capital city of Paramaribo, at four hospitals (the Academic Hospital Paramaribo, Diakonessen Hospital, ‘s-Lands Hospital, Saint Vincentius Hospital) and at prenatal clinics and midwife facilities of the Regional Health Department; (2) the agricultural district Nickerie, at the Mungra Medical Centre Hospital and at Regional Health Department clinics and facilities; and (3) the Amazonian interior, at multiple health care clinics of the Medical Mission Primary Health Care Suriname (MMPHCS). Women were eligible if they were 16 years or older, spoke Dutch, Saramaccan, or Trio, had a singleton gestation, were planning to give birth at one of the study sites and provided written informed consent/assent. A total of 1143 women are included in this study, and data from 743 participants were available at the third trimester study point. Since the interior is a remote and logistically hard to reach area, recruitment of interior women was delayed, resulting in enrollment in the third trimester. Excluded were mothers with miscarriages, stillbirths, multiple gestations, loss to follow-up or those who refused to continue with the study.

### Ethical considerations

This study was approved by the Institutional Review Boards (IRB) of both Tulane University and the Medical Ethical Commission of Suriname’s Ministry of Health (VG 023-14). Potential participants received documentation describing all aspects of the MeKiTamara study (e.g., content, benefits, risks, incentives). All women included in this study (n = 1143) provided written informed consent. Assent was obtained from participants 16 or 17 years of age.

### Data collection

Data for this study were acquired using three self-report questionnaires: Social Support List-Interactions-12 (SSL-I-12), Cohen’s Perceived Stress Scale (PSS), and Edinburgh Depression Scale (EDS). Designated recruiters were trained to administer the questionnaires through face-to-face interviews using encrypted iPads. Data were uploaded in Research Electronic Data Capture (REDCap) training site database for data cleaning and analysis purposes. REDCap is a secure web application for building and monitoring online surveys and databases, and can be used online or offline to collect data for research [28]. Data were collected at two time points: during the first or second trimester of pregnancy (≤ 27 weeks of gestation) and the third trimester of pregnancy (≥ 28 weeks of gestation).

### Questionnaires

The SSL-I-12 was administered once during the first/second trimester, while the PSS and the EDS were both administered twice. As is customary in most similar studies, social support was only measured once, assuming it as an confounding variable with no significant changes during pregnancy [1, 8, 29]. Demographic data were obtained at recruitment and included age, marital status, household income in Surinamese Dollars (SRD), educational level, ethnicity parity, and region. These variables were categorized into the following groups: age (16–19, 20–34 and ≥ 35 years); marital status: married/cohabitating or living alone/single; household income: < 3000 or ≥ 3000 SRD (USD 400); educational level: none, primary or lower secondary versus upper secondary or tertiary; ethnicity: African descent (Creole, Tribal), Asian descent (Hindustani, Javanese), Other (Caucasian, Indigenous, Mixed); parity: no previous live birth, one previous live birth, more than one previous live birth; and region: urban (Paramaribo, Wanica), rural (Commewijne, Saramacca, Para, Nickerie and Coronie), and the interior (Marowijne, Brokopondo and Sipaliwini).

Social support was assessed using the SSL-I-12, which includes twelve statements about support, affection, and attention from family and friends. There are four response options: 1 for rarely or never, 2 for occasionally, 3 for regularly and 4 for very often. Before data collection, one question was deleted due to possible misinterpretation based upon Suriname’s cultural context. Because the SSL-12-I scale was modified, we implemented an exploratory factor analysis, which resulted in a two-factor solution: the Individual Resilience subscale and the Community Engagement subscale (Table 1). A total social support score was calculated by adding the first nine questions together to form the Individual Resilience subscale and the last two for Community Engagement subscale. Only participants who answered all questions were given a total score. The cut-off for low and high were determined by the distribution of the subscales. The median scores for both subscales were used as cut-off points, since the distribution of the subscale scores was skewed.

**Table 1:** Exploratory factor analysis with a two-factor solution

Items	Individual resilience	Community engagement
1 Reassure you?	0.698	
2 Show interest in you	0.669	
3 Give you good advice	0.662	
4 Offer you help	0.650	
5 Comfort you	0.639	
6 Emphasize your strong points	0.597	
7 Are affectionate towards you	0.596	
8 Give you a compliment	0.575	
9 Ask you for help or advice	0.512	
10 Drop in for a (pleasant) visit		0.593
11 Invite you to a party or for dinner		0.508
Eigenvalue	4.470	1.224
% of variance	40.6	11.1
Cronbach α	0.853	0.659

Perceived stress was assessed by the PSS, which contains ten items about the degree of experiencing stress due to having no control over things, nervousness, and not feeling confident about ability to cope with things in the past four weeks. The PSS had an internal consistency (Cronbach α) of 0.676. There are five response options: 0 for never, 1 for almost never, 2 for sometimes, 3 for fairly often and 4 for very often. The total score ranges from 0 (lowest stress level) to 40 (highest stress level) points. The cut-off for high perceived stress was set at 20 points or higher (75th percentile).

The Edinburgh Postnatal Depression Scale assesses postnatal depression, but has been validated for use prenatally. If used prenatally, it is known as the Edinburgh Depression Scale (EDS) [30]. The EDS has been validated in both high and low income settings [31–33], and has been used by several investigators in a number of LMIC settings, including Brazil [34, 35], Jamaica [22], South Africa [36, 37] and Nepal [38]. In addition, it has been validated in Dutch [30, 39], which is the formal language in Suriname and, in line with our inclusion criteria, the questionnaire was thus administered in Dutch. The EDS has a sensitivity of 86%, a specificity of 78% [40], and an internal consistency (Cronbach α) of 0.813. The EDS includes 10 statements concerning anxiety and depression symptoms on a four point Likert scale: 0 = yes, very often; 1 = yes, mostly; 2 = no, not often; and 3 = not at all. A total depression sum score of all statements ranges from 0 to 30 points. A higher total depression score indicates a higher risk of probable depression. In this study, a cut-off point of ≥ 12 points was used to indicate probable depression, compared to a score of 0–11 points for no depression [41]. An exploratory factor analysis was conducted, which resulted in a one-factor solution (Table 2).

**Table 2:** Exploratory factor analysis with a one-factor solution

Items	Factor loadings
1 I have been able to laugh and see the funny side of things	0.491
2 I have looked forward with enjoyment to things	0.479
3 I have blamed myself unnecessarily when things went wrong	0.445
4 I have been anxious or worried for no good reason	0.516
5 I have felt scared or panicky for no very good reason	0.568
6 Things have been getting on top of me	0.580
7 I have been so unhappy that I have had difficulty sleeping	0.720
8 I have felt sad or miserable	0.677
9 I have been so unhappy that I have been crying	0.679
10 The thought of harming myself has occurred to me	0.433
Eigenvalue	3.800
% of variance	38.00
Cronbach α	0.813

### Data analysis

Data were analyzed using IBM SPSS Statistics (version 20). Descriptive statistics were calculated using frequencies and cross tabulation and presented in Table 3. Bivariate logistic regression was used to determine the association between social-demographics and PSS (Table 3), and between PSS and EDS and presented as unadjusted odds ratio (OR) with 95% confidence intervals (CI) and p-values. Paired t-tests (Table 4) were performed to compare the means of PSS and EDS during the first visit and second prenatal visit. Multivariate logistic regressions for depression during first/second trimester and third trimester were conducted, adjusted for socio-demographics that were significantly associated with PSS (adjusted ORs (AOR), CIs and p-values are presented) (Table 5). A *p*-value ≤ 0.05 was considered significant.

**Table 3:** Characteristics of the study population

Characteristics 1st/2nd trimester	Total n (%)	High perceived stress score 20–40 n (%)	Low perceived stress score 0–19 n (%)	COR (95% CI)	*p*-value
Perceived stress 1st/2nd trimester	1143	304 (27.2)	812 (72.8)		
Perceived stress 3rd trimester	743	181 (24.7)	553 (75.3)		
Social support					**0.001**
Community engagement					
Low	628 (54.9)	194 (31.6)	419 (68.4)	1.67 [1.27–2.19]	
High	513 (44.9)	109 (21.8)	392 (78.2)	1	
Missing	2 (0.2)				
Individual resilience					
Low	595 (52.1)	184 (32.1)	389 (67.9)	1.75 [1.33–2.29]	
High	548 (47.9)	113 (21.3)	417 (78.7)	1	
Age (Years)					**0.036**
Mean (SD)	28 (6.43)				
16–19	144 (12.6)	50 (36.5)	87 (63.5)	**1.64 [1.12–2.41]**	
20–34	819 (71.7)	208 (25.9)	595 (74.1)	1	
35^+^	179 (15.7)	46 (26.3)	129 (73.7)	1.02 [0.70–1.48]	
Missing	1 (0.1)				
Ethnicity					**0.001**
African descent	520 (45.5)	163 (32.3)	342 (67.7)	**1.93 [1.36–2.74]**	
Asian descent	334 (29.2)	85 (25.8)	244 (74.2)	1.41 [0.96–2.08]	
Other	285 (24.9)	55 (19.8)	223 (80.2)	1	
Missing	4 (0.3)				
Income (SRD)					**0.012**
< 3000	763 (66.8)	217 (29.1)	529 (70.9)	1.48 [1.09–2.01]	
≥ 3000	333 (29.1)	71 (21.7)	256 (78.3)	1	
Missing	47 (4.1)				
Educational level					**0.001**
None, primary, lower secondary/vocational	658 (57.6)	206 (32.2)	434 (67.8)	1.83 [1.39–2.42]	
Upper secondary/vocational or tertiary	485 (42.4)	98 (20.6)	378 (79.4)	1	
Missing	0 (0.0)				
Marital status					0.065
Married/cohabitating	1000 (87.5)	257 (26.3)	719 (73.7)	1	
Not married/not living together	141 (12.3)	47 (33.8)	92 (66.2)	1.43 [0.98–2.09]	
Missing	2 (0.2)				
Parity					0.459
0 (primiparity)	384 (33.6)	108 (28.8)	267 (71.2)	1	
1	312 (27.3)	76 (24.7)	232 (75.3)	0.81 [0.58–1.14]	
≥ 2	445 (38.9)	120 (27.8)	311 (72.2)	0.95 [0.70–1.30]	
Missing	2 (0.2)				
Region					0.074
Urban	656 (57.4)	191 (29.6)	454 (70.4)	**1.47 [1.05–2.05]**	
Rural	276 (24.1)	60 (22.3)	209 (77.7)	1	
Interior	211 (18.5)	53 (26.2)	149 (73.8)	1.24 [0.81–1.90]	

Bold indicates significance

**Table 4:** Comparison of means of perceived stress and depression during pregnancy

	1st/2nd trimester Mean (SD)	3rd trimester Mean (SD)	95% CI	*p*-value
Perceived stress (n = 718)	16.02 (5.22)	15.70 (5.11)	− 0.06–0.71	0.098
Depression (n = 720)	7.80 (5.07)	6.93 (4.56)	0.54–1.21	**0.001**

Bold indicates significance

**Table 5:** Multivariate logistic regression of socio-demographic factors and depression during 1st/2nd and 3rd trimesters of pregnancy

Variables	1st/2nd trimester		3rd trimester	
	AOR (95% CI)	*p*-value	AOR (95% CI)	*p*-value
Social support				
Community engagement		0.914		0.501
Low	1.02 [0.74–1.40]		1.17 [0.74–1.87]	
High	1		1	
Missing				
Individual resilience		**0.027**		**0.038**
Low	1.45 [1.04–2.01]		1.65 [1.03–2.63]	
High	1		1	
Perceived stress (1st/2nd trimester)		**0.001**		**0.008**
Low	1		1	
High	7.21 [5.15–10.09		1.92 [1.18–3.11]	
Perceived stress (3rd trimester)	–	–		**0.001**
Low			1	
High			7.48 [4.64–12.05]	
Age (Years)		0.135		0.072
16–19	0.59 [0.35–0.99]		1	
20–34	1		**3.14 [1.17–8.41]**	
35^+^	0.88 [0.55–1.40]		2.64 [0.87–8.03]	
Ethnicity		0.913		0.631
African descent	1.00 [0.65- 1.53]		0.72 [0.37- 1.42]	
Asian descent	0.91 [0.56–1.49]		0.85 [0.42–1.72]	
Other	1		1	
Income (SRD)		0.449		0.383
< 3000	1.18 [0.77–1.79]		1.30 [0.72–2.36]	
≥ 3000	1		1	
Educational level		**0.007**		**0.004**
None, primary, lower secondary/vocational	1.72 [1.16–2.55]		2.23 [1.29–3.86]	
Upper secondary/vocational or tertiary	1		1	
Marital status		**0.044**		0.962
Married/cohabitating	1		1	
Not married/not living together	1.65 [1.01–2.69]		1.02 [0.50–2.06]	
Region		0.990		0.210
Urban	1		1	
Rural	1.01 [0.64–1.58]		0.62 [0.33–1.15]	
Interior	1.04 [0.64–1.68]		0.66 [0.31–1.38]	

Bold indicates significance

## Results

Table 3 shows the demographic characteristics of the study population. The average age was 28 years (SD 6.43) with a range of 16–49 years. Most participants were 20–34 years old (71.7%), were of African descent (45.5%), had household income < 3000 SRD (66.8%), and were lower educated (57.6%), and had ≥ 2 previous live births (38.9%). The majority were married/cohabitating (87.5%) and lived in urban areas (57.4%). Most participants experienced social support levels below the median, indicating low social support. 54.9% of participants scored below the median for community engagement and 595 (52.1%) for individual resilience. High perceived stress occurred in 27.2% of the participants during the first/second trimester and 24.7% during the third trimester of pregnancy. Probable depression was identified in 22.4% of the participants during the first/second trimester and in 17.6% during third trimester.

Bivariate regression analyses showed statistically significant associations between social support, age, ethnicity, income, educational level, region, and high perceived stress (Table 3). Pregnant women who scored low for community engagement had a higher likelihood (OR 1.67; 95% CI 1.27–2.19) for high perceived stress. Similarly, women who scored low on individual resilience had a higher risk (OR 1.75; 95% CI 1.33–2.29) for high perceived stress. Women aged 16–19 years (OR 1.64; 95% CI 1.12–2.41), of African descent (OR 1.93; 95% CI 1.36- 2.74), and with lower household income (OR 1.48; 95% CI 1.09- 2.01) had higher perceived stress levels compared to women 20–34 years, who were Caucasian, Indigenous and Mixed, and with higher household incomes. Similarly, women with lower education (OR 1.83 95% CI 1.39–2.42) and living in urban areas (OR 1.47 95% CI 1.05–2.05) perceived significantly higher stress levels compared to higher educated women living in rural areas. Marital status (*p* = 0.065) and parity (*p* = 0.459) were not associated with high perceived stress.

During the first/second trimester, 145 (48.8%) women with high perceived stress levels had probable depression. These women had 6.82 increased odds (95% CI 5.00–9.31) of having probable depression compared to women with low perceived stress levels. During the third trimester, 82 (45.8%) women experienced high perceived stress levels. These women had 9.85 times the odds (95% CI 6.42–15.12) of having probable depression compared to women with low perceived stress levels.

A decrease in mean perceived stress (from 16.02 to 15.70; *p* = 0.098) and probable depression (7.80–6.93; *p* = 0.001) was noted between the first/second and the third trimester (Table 4). The decrease in probable depression was statistically significant, but not the decrease in perceived stress.

Results of multivariate logistic regression to assess the association between socio-demographic factors and depression during the first/second and third trimester appear in Table 5. Statistically significant associations remained between perceived stress, educational level, and marital status, and probable depression. During first/second trimester, women who perceived high stress levels (OR 7.21 95% CI 5.15–10.09), were lower educated (OR 1.83 95% CI 1.39–2.42), and were unmarried/single, (OR 1.65 95% CI 1.01–2.69) were at higher risk of probable depression compared to women with low stress, a higher education, and who were married/cohabitating. Women who scored low for individual resilience had 1.45 (95% CI 1.04–2.01) increased odds of having high perceived stress levels compared to women who scored high.

During third trimester, participants 20–34 years old had a threefold higher risk (95% CI 1.17–8.41) of having probable depression compared to those 16–19 years of age. Lower educated women had twice the odds (95% CI 1.29–3.86) of experiencing probable depression than higher educated women. Women who experienced high stress levels had 7.48 (95% CI 4.64–12.05) the odds of having probable depression compared to women with low stress levels. Participants with low scores for individual resilience had 1.65 (95% CI 1.03–2.63) the odds of having high perceived stress levels compared to women who scored high for individual resilience. Women experiencing high stress levels during the first/second trimester had twice the odds of having probable depression during the third trimester (*p* = 0.008). Ethnicity, household income, marital status and region were not significantly associated with third trimester depression.

## Discussion

One out of five pregnant women enrolled in the MeKiTamara study in Suriname suffered from probable depression throughout pregnancy. Approximately one in four participants experienced high perceived stress levels, which in turn was significantly associated with probable depression during both early and late pregnancy. Participants with low social support or a lower education level had increased risk of depression throughout pregnancy. Participants aged 20 to 34 years had more chance of probable depression during the third trimester than women aged 16–19 years.

Studies conducted in Ethiopia, Malaysia and Brazil reported similar prevalence of prenatal depression at 21.5%, 20% and 19.6% respectively [41–43]. However, not all of these studies reported prevalence according to pregnancy trimester, therefore limiting comparison [41–43]. A study of 5301 multi-ethnic and socioeconomically diverse women in New Zealand identified 16.5% prenatal depression in the third trimester; in line with our results of 17.6% in the third trimester [28], despite the geographical, cultural and economic differences between New Zealand and Suriname. In contrast to our study, however, educational level was not significantly associated with depression in the New Zealand study. This difference may be explained by the fact that more MeKiTamara participants had no or lower education than those from New Zealand. A Norwegian study with 33,774 participants revealed that high educational level has a protective effect on mental health. This effect accumulates throughout life, indicating that older, educated pregnant women would have lower risk of depression [44]. This contradicts our study where older women had higher odds of depression than younger women during the third trimester of pregnancy. Again, this may be explained by the fact that most MeKiTamara participants (57.6%) had lower educational levels.

Reports on the course of depression during pregnancy differ. Gavin et al. systematic review describes a decrease in the prevalence of depression from 11% in the first trimester to 8.5% in the third trimester [16], while Ayano et al.’s systematic review and meta-analysis mention a higher prevalence of prenatal depression during third trimester compared to the first and second trimester [15]. Among MeKiTamara participants, data indicate that both a decrease in perceived stress during the third trimester compared to the first two trimesters and an increase in perceived social support during the third trimester may have contributed to less depression in the last trimester of pregnancy. We recommend further research on this.

In terms of stress, a Saudi Arabian study of 438 pregnant women during all trimesters of pregnancy reported 33.4% high perceived stress levels using the PSS [1]. In contrast, an Ethiopian study among 396 pregnant women using the PSS-7 found a high perceived stress level of 11.6% during pregnancy [2]. Rates for high perceived stress of approximately 26% among MeKiTamara participants falls between these two studies. These differences in prevalence may be due to the homogeneous nature of the other studies’ participants with respect to race and ethnicity, residence, access to care and other social determinants of health. Whereas these studies mainly included participants from one or a few hospitals, MeKiTamara included participants from different regions with more than 40 study locations. The dissimilarity in the prevalence of high perceived stress could also be explained by differences in socio-demographic variables as educational level, income levels and employment status, cultural differences and geographical differences across the three studies. Low individual resilience, which in turn was significantly associated with perceived stress and depression throughout pregnancy, may also explain MeKiTamara’s higher prevalence for perceived stress and depression compared to Ethiopia [2], and the lower prevalence of depression in Brazil. [43] Finally, one possible explanation for the low prevalence of high perceived stress in the Ethiopian study was that most of the pregnant women were living with their partner. This is consistent with our finding that women who were married or co-habitating had a lower risk for depression.

From a public health practice perspective, the high levels of perceived stress and depression in this study call for effective, timely prenatal screening of perceived stress and depression by general practitioners, gynecologists, or midwives at regular prenatal visits. Also, involving partners in prenatal visits may improve social support for pregnant women. Moreover, utilizing community health workers (CHWs) to link pregnant women to mental health care could lower barriers to care, especially among vulnerable groups, e.g. pregnant women. Health education programs, targeting the reduction of stress during pregnancy, may help to reduce depression and its potential adverse health effects on the mother and child.

### Strengths and limitations

The strength of the study is multi-pronged. To our knowledge, this is the first study in Suriname to assess the influence of perceived stress, social support, and demographic variables on prenatal depression. The large sample size (n = 1143) and the geographic diversity of our study population boosts external validity. In addition, the ethnic and cultural diversity of the study population, combined with the range of socio-demographic factors, enhances potential generalizability.

Among the limitations is that the questionnaires used in this study, although standardized and previously used in LMICs, were not specifically validated for Suriname before data collection. However, explanatory factor analysis indicated high factor loadings on that factor(s) and no cross loadings. Given these findings it would be appropriate to use these scales in Suriname. In this study we ideally would include participants who were in their first trimester of pregnancy, but many participants, especially those living in the interior amazon rainforest, did not avail themselves of prenatal care until early in the 2nd trimester of pregnancy. This means that we have data of one first study time point, either the first or the second trimester of pregnancy. Perceived stress and probable depression were measured with a screening tool and not clinically assessed by a mental health specialist. Thus, the prevalence of probable depression may not exactly correspond with the actual prevalence of depression. Still, this is a minor limitation since the EDS questionnaire does not produce artificially high scores [45]. Furthermore, a common limitation of previous studies is inherent to the use of EDS as a screening tool for depression since this tool partially measures anxiety. It was therefore not possible to consider the role of anxiety in these studies, including this study. Symptoms of mental distress are often interwoven and it is possible that the aspects of EDS that relate to anxiety are partly measuring similar constructs relative to stress.

Finally, as is customary in most studies, social support was only measured once, assuming no significant changes during pregnancy. However, this assumption may not always be correct—divorce, moving away from family for work, and domestic violence can all impact social support. Ascertaining social support both prenatally and postnatally may provide a better assessment not only of the level of support, but also how the changing degree of support impacts stress and depression.

## Conclusions

This first study to examine the influence of perceived stress and social support on prenatal depression in Suriname makes a significant contribution to public health science and has implications for prenatal care in the country. Further research is needed to examine other risk factors for depression during pregnancy, such as previous history of depression, unintended pregnancies, and domestic violence. In addition, it is pivotal to evaluate the influence of perceived stress, low social support, and depression during pregnancy on birth outcomes in Suriname.

## Data Availability

The datasets generated and/or analysed during the current study are not publicly available due to ongoing data analysis beyond what is currently included in this study but are available from the corresponding author on reasonable request.

## Abbreviations

CCREOH: Caribbean Consortium for Research in Environmental and Occupational Health
LMICs: Low and Middle Income Countries
CRP: C-reactive protein
MMPHCS: Medical Mission Primary Health Care Suriname
IRB: Institutional Review Boards
SSL-I-12: Social Support List-Interactions-12
PSS: Cohen’s Perceived Stress Scale
EDS: Edinburgh Depression Scale
SRD: Surinamese Dollars
REDCap: Research Electronic Data Capture
